# Effects of different drying techniques of ground sprouted chickpeas on quality, textural properties, and sensory attributes of fried falafel

**DOI:** 10.1002/fsn3.4240

**Published:** 2024-06-17

**Authors:** Kimia Goharpour, Fakhreddin Salehi, Amir Daraei Garmakhany

**Affiliations:** ^1^ Department of Food Science and Technology, Faculty of Food Industry Bu‐Ali Sina University Hamedan Iran; ^2^ Department of Food Science and Technology, Toyserkan Faculty of Engineering and Natural Resources Bu‐Ali Sina University Hamedan Iran

**Keywords:** antioxidant capacity, hot‐air, infrared, microwave, phenolic content

## Abstract

The process of sprouting makes legumes better by changing their nutrition, chemicals, and taste. Ground sprouted chickpeas are commonly used as the main ingredient in falafel. The aim of this research was to study the impact of drying techniques that include hot‐air, infrared, and microwave on the moisture, ash, total phenolics, antioxidant capacity, color, and rehydration ratio of dried ground sprouted chickpeas. Also, the effects of drying techniques of ground sprouted chickpeas on the moisture, ash, total phenolics, antioxidant activity, color, volume, density, oil content, textural properties, and sensory attributes of fried falafel were examined. The total phenolics of hot‐air‐dried, infrared‐dried, and microwave‐dried samples were 463.42, 766.20, and 470.82 μg Gallic acid (GA)/g dry, respectively. The infrared‐dried ground sprouts had the highest antioxidant capacity. Additionally, the total phenolic content and antioxidant capacity of fried falafels made from infrared‐dried ground sprouted chickpeas were significantly higher than those of the fried falafels made from hot‐air‐dried and microwave‐dried sprouts (*p* < .05). The mean values of lightness, redness, and yellowness of the fried falafel core ranged from 57.37 to 59.29, 3.66 to 6.88, and 40.50 to 43.31, respectively. The lowest oil absorption was related to the sample prepared from the hot‐air‐dried sprouted chickpeas (*p* < .05). The firmness, cohesiveness, springiness, and chewiness of the fried falafels were between 38.17 N and 91.56 N, 0.29 and 0.44, 0.48 and 0.58, and 5.38 N and 17.24 N, respectively. The use of infrared‐dried sprouted chickpeas for producing fried falafel, due to the highest odor, flavor, and overall acceptance scores, high phenolic compounds and antioxidant activity, high volume, low density, and low hardness, is recommended.

## Graphical abstract

The purpose of this research was to study the impact of drying techniques that include hot‐air, infrared, and microwave on the moisture, ash, total phenolics, antioxidant capacity, color, and rehydration ratio of dried ground sprouted chickpeas. Also, the effects of drying techniques of ground sprouted chickpeas on the moisture, ash, total phenolics, antioxidant activity, color, volume, density, oil content, textural properties, and sensory attributes of fried falafel were examined.

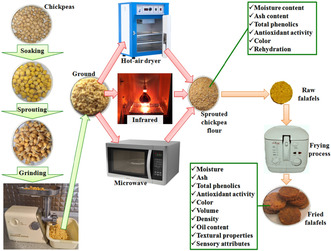

## 1 INTRODUCTION

Chickpeas (*Cicer arietinum* L.) are grown in many different climates and are the third most important legume crop in the world after soybean and bean (Bidkhori & Mohammadpour Karizaki, 2022; Doddamani et al., 2014; Ghoshal & Kaushal, 2020).

The most common methods for drying agricultural products are the use of hot‐air flow and displacement heat transfer methods, which involve the simultaneous transfer of mass and heat within the product. Compared to other modern methods, hot‐air drying has advantages such as the ability to accurately control temperature and process conditions, but disadvantages such as longer process time and lower product quality (Mujumdar, 2014). One way to reduce the drying time and improve dry product quality is to use infrared radiation. Infrared technology is highly energy‐efficient, less water‐consuming, and environmentally friendly compared to conventional heating. The use of infrared radiation increases the drying rate and maintains the quality of the final product (Aboud et al., 2019; Salehi, 2020). Nachaisin et al. (2016) used a combined infrared‐hot air method to dry instant germinated brown rice. Based on the results reported in this research, the use of infrared radiation increased the drying rate and decreased the drying duration. Also, the energy consumption decreased with increasing radiation intensity. Su et al. (2020) have proposed the combination of sprouting and infrared drying as a suitable method to modify the properties of naked barley flour and starch to promote their use in the food industry. Microwaves are a fast and effective heating source that directly affects the whole food product, accelerating physicochemical reactions and the drying rates to produce high‐quality dry products (Wray & Ramaswamy, 2015). The results of reports related to various studies have shown that using microwave pretreatment causes less damage to the nutritional components of plants (Akbarian Meymand et al., 2015). Bualuang et al. (2017) investigated the influence of microwave drying on the quality of germinated corn. The finding of this research confirmed that drying with microwaves (power 300 W) preserves the nutritional value of dried sprouted corn and increases its antioxidant activity.

The human being has historically consumed fried foods for centuries (Coria‐Hernández et al., 2023). All over the world, the development of products that resemble meat but contain predominantly plant‐sourced ingredients is a prime focus (Moonaisur et al., 2024). Falafel is a traditional food that consists of chickpea flour, water, onion, garlic, salt, pepper, sesame, and various spices (Ismail & Kucukoner, 2017). Falafels are fried in hot oil and uptake a lot of oil, which poses potential risks and dangers to human health when consumed continuously (Hojjati et al., 2020).

For processing and drying each product, it is necessary to use the optimal drying method under optimal conditions in order to minimize deterioration in the quality of the target product and complete the operation in the shortest possible time (Khodadadi et al., 2017, 2023). So, the aim of this work was to examine the influence of drying techniques that include hot‐air, infrared, and microwave on the physicochemical characteristics of dried ground sprouted chickpeas. As well, the effects of drying techniques of ground sprouted chickpeas on the quality of fried falafel were examined.

## 2 MATERIALS AND METHODS

### 2.1 Sprouting process

**FIGURE 1:**
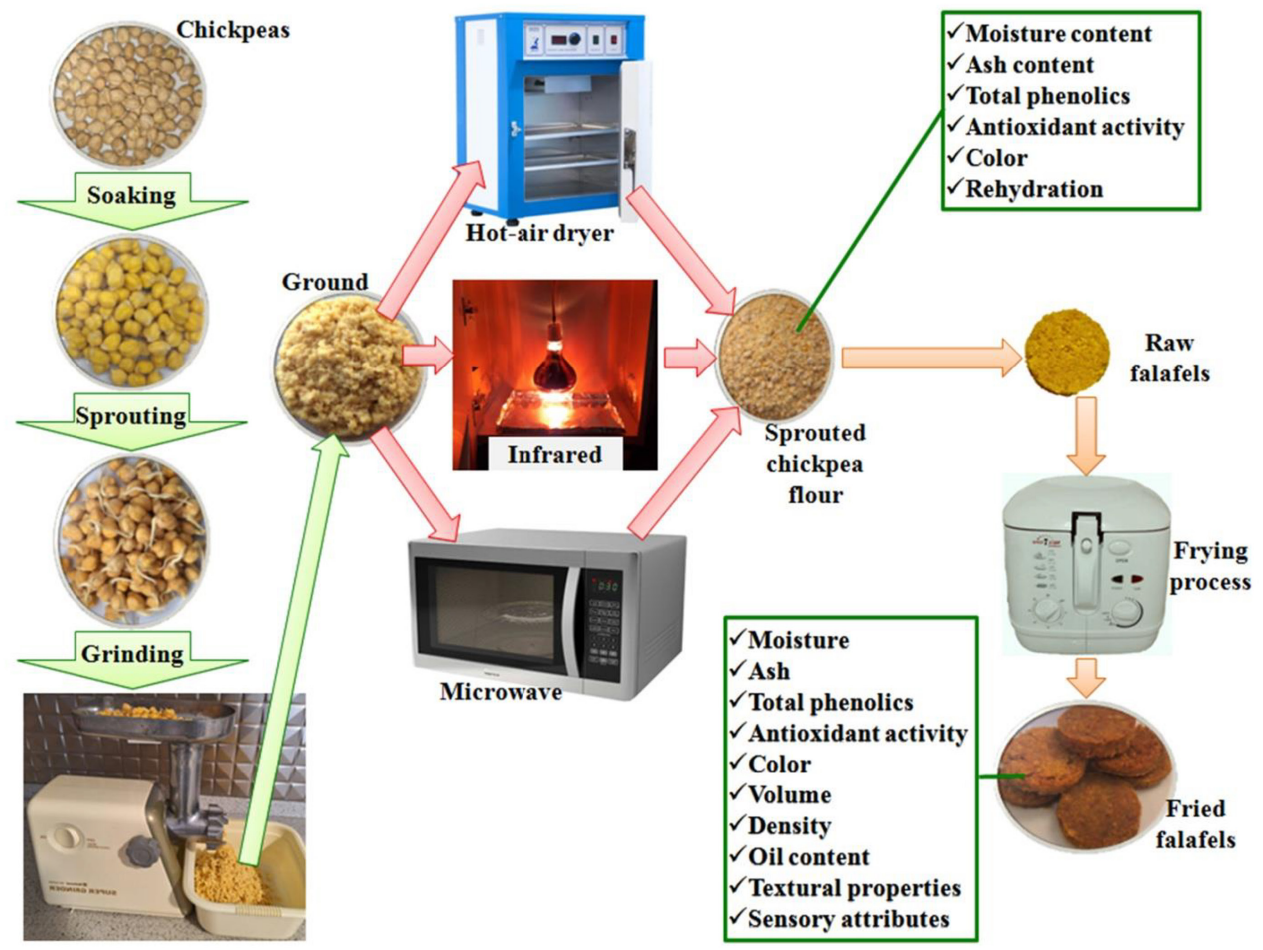
Drying of ground sprouted chickpeas using hot‐air, infrared, and microwave dryers for producing fried falafel.

After washing the purchased chickpeas with water, they were soaked in water for 24 h at 25°C. Afterward, excess water was completely removed, and sprouting of chickpeas was carried out at 25°C for 24 h in a container covered with a thin towel (the water in the samples was changed every 6 h) (Salehi, 2023; Su et al., 2020). The sprouted chickpeas were ground using a meat grinder (MK‐G20NR, National, Japan) (Figure 1).

### 2.2 Drying techniques (hot‐air, infrared, and microwave)

To dry the samples, 250 g ground sprouted chickpeas were poured as a thin layer into aluminum containers and placed in hot‐air (at 70°C, Shimaz), infrared (250 W, near‐infrared), and microwave (220 W, Gplus, GMW‐M425S.MIS00, Goldiran Industries Co.,) dryers, until the samples reached a constant weight. The dried ground sprouted chickpeas were cooled and poured into a polyethylene bag (moisture proof), sealed, and stored in a refrigerator (5–7°C).

### 2.3 Preparation of fried falafel

The falafel recipe prepared included dried ground sprouted chickpeas (140 g), water (100 g), salt (3.5 g), falafel spice (1.1 g), and baking powder (1 g). For this research, we purchased liquid oil from Oila company (frying oil), salt from Toloo company, baking powder from Bartar company, and falafel spice powder from a store in Hamedan. After the raw materials (ingredients) were weighed (using a digital scale, 200 g/0.01 g, with a sensitivity of ±0.01 g), they were mixed thoroughly by hand. After 15 min, the prepared mixture was evenly distributed using a mold and transferred to a fryer (Seven star, Model df02) at 150°C for 8 minutes. Finally, the fried falafels were removed from the fryer, cooled, and stored in polyethylene bags.

### 2.4 Moisture and ash contents

In this research, the moisture contents of dried ground sprouted chickpeas and fried falafels were measured by an oven at 105°C for 5 h (Shimaz) (Hosseini, 2006). At this stage, the mass changes of the samples were recorded using a laboratory scale (±0.01 g, Lutron GM‐300P).

The ash contents of dried ground sprouted chickpeas and fried falafels were measured using a laboratory electric furnace at 550°C for 6 h (Hosseini, 2006). The ash content (%) of the samples was calculated using Equation 1.

(1) $$\mathrm{Ash}\;\left(\%\right)=\left(\frac{M2-M3}{M1}\right)\times 100$$

In this equation, *M*_1_ is the initial weight of the sample (3 g), *M*_2_ is the weight of the sample + crucible after the furnace, and *M*_3_ is the weight of the empty crucible. At this stage, the sample weight was recorded using a laboratory balance (Sartorius, Switzerland) with a sensitivity of ±0.001 g.

### 2.5 Total phenolic contents

To prepare the sample extract, 10 mL of 80% methanol was added to 1 g of dried ground sprouted chickpeas and fried falafels and mixed with a magnetic stirrer (hot plate stirrer, Shimaz) for 30 min. After this step, the mixture was transferred to the falcon tube. The falcon tube was centrifuged for 5 min at a speed of 4000 rpm (revolutions per minute) using a Centrifuge device (Universal 320R, Hettich). The supernatant of the mixture was then used as the extract. The total phenolic content of samples was estimated as Gallic acid equivalent (GAE) using Folin–Ciocalteu's phenol reagent (Sigma‐Aldrich) and a ultraviolet–visible (UV–VIS) spectrophotometer at 725 nm (XD‐7500, Lovibond) (Salehi, Ghazvineh, & Inanloodoghouz, 2023).

### 2.6 Antioxidant capacity

The antioxidant capacity of dried ground sprouted chickpeas and fried falafels was estimated as free radical‐scavenging activity using DPPH (2,2‐diphenyl‐1‐picrylhydrazyl, 0.1 mM, Sigma‐Aldrich, USA) and a UV–VIS spectrophotometer at 517 nm (XD‐7500, Lovibond) (Salehi, Ghazvineh, & Inanloodoghouz, 2023).

### 2.7 Rehydration ratio

The rehydration ratio tests of dried ground sprouted chickpeas were conducted using a water bath (R.J42, Pars Azma Co.) at 50°C for 30 min (Salehi, Razavi Kamran, & Goharpour, 2023).

### 2.8 Color measurement

The dried ground sprouted chickpeas and fried falafels photographs were taken using a scanner (HP Scanjet‐300). Using a computer program (ImageJ, V.1.42e) and its color space conversion plug‐in, the samples’ photographs were converted from red, green, and blue (RGB) color space to L*a*b* color space (L*: darkness/lightness, a*: greenness/redness, and b^*:^ blueness/yellowness) (Chatchavanthatri et al., 2021; Salehi, 2019).

### 2.9 Volume and density of fried falafels

The volume of the fried falafels was estimated by the canola displacement technique. The volume of fried falafels was averaged from three replications. The fried falafel mass was recorded by a laboratory balance (±0.01 g, Lutron GM‐300P, Taiwan). The density of fried falafels was calculated using Equation 2.

(2) $$\rho \;\left(\mathrm{kg}/m^{3}\right)=\frac{M}{V}$$

In this equation, *ρ* is the density of the fried falafels (kg/m^3^), M is the mass of the fried falafels (kg), and V is the volume of the fried falafels (m^3^).

### 2.10 Oil content (Soxhlet extraction)

Conventional Soxhlet extraction was performed using 3 g of fried falafels and ∼ 75 mL of n‐Hexane (extra pure, Arman sina) in a laboratory Soxhlet Extractor (Behr E4) at 80°C for 5 h.

### 2.11 Textural properties

The texture determination of the fried falafels was performed on a STM‐5 texture analyzer (Santam). The texture of the fried falafels was measured when their surface temperature was cooled up to 25°C. A puncture test was performed using a cylindrical flat‐end punch (diameter 5 mm).

The test parameters were: pre‐test and test speed, 1 mm/s and travel distance, 10 mm. The test was repeated three times (Li et al., 2022). Texture profile analysis (TPA) of fried falafel samples (a cylindrical shape with a diameter of 3.5 cm and a thickness of 0.8 cm) was carried out in two compression steps (using a 5 cm diameter cylindrical probe) at the pre‐test, test, and post‐test speeds of 1, 1, and 1 mm/s. The compression distance was 50% strain (Chatchavanthatri et al., 2021).

### 2.12 Sensory evaluation

A hedonic test was used to estimate the general level of liking for the fried falafels. Twenty‐one panelists (the ages of 17–55 years) were selected and recruited to descriptively analyze the texture of fried falafels. In the sensory evaluation of fried falafels, 21 panelists were given three samples and rated their level of fried falafels liking according to a hedonic 9‐point scale (1 = dislike extremely, 5 = neither like nor dislike, 9 = like extremely) (Tukassar et al., 2023).

### 2.13 Statistical analysis

In this research, the experimental results were expressed as means ± standard deviations. All the experiments were performed in triplicate. Statistical significance was established by an analysis of variance (ANOVA) using statistics software (SPSS 21). A value of *p* < .05 was regarded as statistically significant (Chen et al., 2024).

## 3 RESULTS AND DISCUSSION

### 3.1 Physicochemical properties of dried ground sprouted chickpeas

In this research, the moisture and ash contents of unsoaked chickpeas were 6.89% and 3.40%, respectively. Table 1 shows the effects of drying techniques on moisture content, ash content, antioxidant capacity, and rehydration ratio of the ground sprouted chickpea. The moisture contents of hot‐air‐dried, infrared‐dried, and microwave‐dried ground sprouted chickpeas were 7.80%, 5.80%, and 7.76%, respectively. In addition, the ash contents of hot‐air‐dried, infrared‐dried, and microwave‐dried ground sprouted chickpeas were 2.37%, 2.46%, and 2.53%, respectively. The findings demonstrated that there was no statistically significant change in the mean values of ash contents of dried sprouts.

**TABLE 1:** Impact of drying techniques on moisture, ash, antioxidant capacity, and rehydration of the ground sprouted chickpea.

Drying method	Moisture content (%)	Ash content (%)	Antioxidant capacity (%)	Rehydration ratio (%)
Hot‐air	7.80 ± 0.43^a^	2.37 ± 0.03^a^	23.97 ± 1.87^c^	286.67 ± 10.73^a^
Infrared	5.80 ± 0.16^b^	2.46 ± 0.15^a^	91.61 ± 0.15^a^	260.60 ± 2.29^b^
Microwave	7.67 ± 1.20^a^	2.53 ± 0.12^a^	67.61 ± 0.79^b^	302.00 ± 3.93^a^

*Note*: Data are shown as mean ± standard deviation (*N* = 3). According to the one‐way ANOVA and the Duncan post hoc test, different letters within columns indicate means that are significantly different at *p* < .05.

**FIGURE 2:**
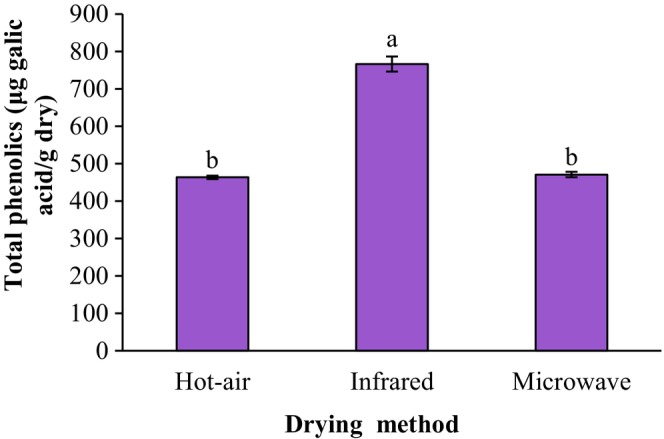
Impact of drying techniques on total phenolics contents of the ground sprouted chickpeas. Data are shown as mean ± standard deviation (*N* = 3). According to the one‐way ANOVA and the Duncan post hoc test, different letters above the columns indicate significant differences (*p* < .05).

Figure 2 shows the effects of drying techniques on total phenolic contents of the ground sprouted chickpeas. The total phenolics of hot‐air‐dried, infrared‐dried, and microwave‐dried samples were 463.42, 766.20, and 470.82 μg GA/g dry, respectively. Since the drying time of the ground sprouts in the infrared‐dryer was shorter in the hot‐air and microwave dryers (26.7 min versus 63.3 min and 156.7 min, respectively), more phenolic compounds remained in this state and the amount of these compounds in the prepared flour was also higher. Preservation of phenolic compounds during the drying process of sprouts increases the antioxidant capacity of the dried product. In this study, the antioxidant capacity (free radical‐scavenging activity) of hot‐air‐dried, infrared‐dried, and microwave‐dried ground sprouted chickpea was 23.97%, 91.61%, and 67.61%, respectively. The infrared‐dried ground sprouts had the highest antioxidant capacity, and the hot‐air‐dried sample had the lowest antioxidant capacity. Aboud et al. (2019) reported that total phenolics in infrared‐dried orange peels were higher than in hot‐air oven‐dried orange peels, as infrared reactivated the low‐molecular‐weight antioxidants. Irakli et al. (2018) reported that infrared heat treatment of rice bran increased the phenolic content and antioxidant activity of the stabilized combined extract of rice bran.

Because the drying duration of the ground sprouts in the infrared‐dryer was shorter than those of the ground sprouts in the hot‐air and microwave dryers, the rehydration ratio of dried sprouts in this dryer was low. As shown in Table 1, the microwave‐dried ground sprouts had the highest rehydration ratio (302.0%), and the infrared‐dried sample had the lowest rehydration ratio (260.6%). Also, sufficient energy transmission and water migration under microwave drying partially damaged starch granules and more hydrophilic groups were exposed (Su et al., 2020); therefore, the microwave‐dried ground sprouted chickpea showed greater rehydration than the infrared‐dried sample.

Table 2 shows the drying techniques on color parameters (lightness (L*), redness (a*), and yellowness (b*)) of the ground sprouted chickpeas. Among the dried samples, the dried ground sprouts with infrared radiation had the lowest lightness (63.03), and the highest redness (14.03) and yellowness (31.37) indexes. The infrared energy could effectively penetrate into and heat up the ground sprouted chickpeas, leading to a more browning reaction. Therefore, the samples dried with infrared radiation were redder and yellower than the other samples. The change in color of infrared‐dried samples might be due to the higher caramelization and browning reactions. Additionally, the enzymatic and nonenzymatic browning at high‐temperature drying led to the formation of a brown pigment, which further reduced the lightness (Kumar et al., 2020). Irakli et al. (2018) reported that infrared heat treatment of rice bran reduced lightness due to the formation of brown polymers as a result of the infrared‐induced Maillard reaction.

**TABLE 2:** Impact of drying techniques on color parameters of the ground sprouted chickpeas.

Drying method	Lightness	Redness	Yellowness
Hot‐air	66.02 ± 0.94^ab^	10.94 ± 0.30^b^	28.93 ± 0.60^b^
Infrared	63.03 ± 3.02^b^	14.03 ± 0.90^a^	31.37 ± 0.41^a^
Microwave	69.90 ± 2.14^a^	10.32 ± 0.58^b^	28.49 ± 0.76^b^

*Note*: Data are shown as mean ± standard deviation (*N* = 3). According to the one‐way ANOVA and the Duncan post hoc test, different letters within columns indicate means that are significantly different at *p* < .05.

### 3.2 Moisture and ash contents of fried falafels

**FIGURE 3:**
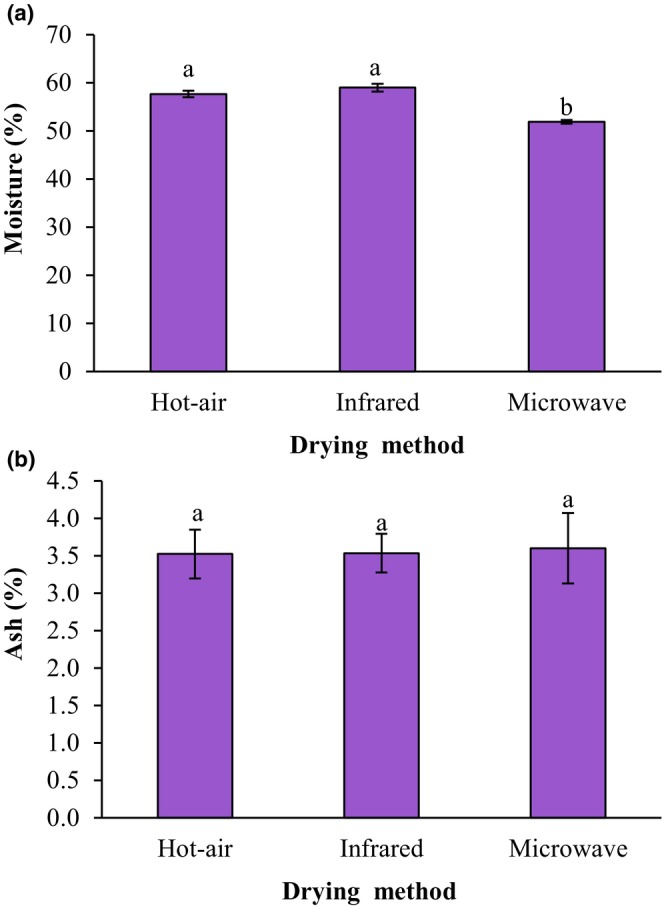
Impact of drying techniques of the ground sprouted chickpeas on moisture (a) and ash (b) contents of fried falafel. Data are shown as mean ± standard deviation (*N* = 3). According to the one‐way ANOVA and the Duncan post hoc test, different letters above the columns indicate significant differences (*p* < .05).

Figure 3a displays the influence of drying techniques of the ground sprouted chickpeas on the moisture content of fried falafel samples. The moisture contents of fried falafel samples prepared from hot‐air‐dried, infrared‐dried, and microwave‐dried ground sprouted chickpeas were 57.67%, 59.00%, and 51.87%, respectively. The moisture content of fried falafel made from infrared‐dried chickpea flour was significantly higher than that of microwave‐dried flour (*p* < .05).

Figure 3b displays the influence of drying techniques of the ground sprouted chickpeas on the ash content of fried falafel samples. In terms of ash content, there was no statistically significant change between fried falafel samples (*p* > .05). The ash contents of fried falafel samples prepared from hot‐air‐dried, infrared‐dried, and microwave‐dried ground sprouted chickpeas were 3.52%, 3.53%, and 3.60%, respectively.

### 3.3 Total phenolics content and antioxidant capacity of fried falafels

**FIGURE 4:**
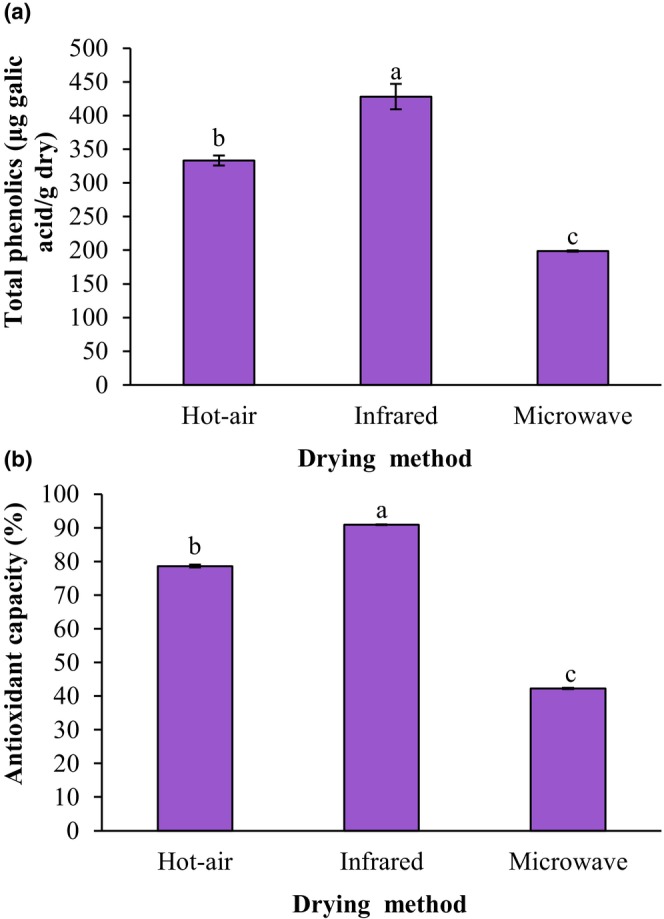
Impact of drying techniques of the ground sprouted chickpeas on total phenolics contents (a) and antioxidant capacity (b) of fried falafel. Data are shown as mean ± standard deviation (*N* = 3). According to the one‐way ANOVA and the Duncan post hoc test, different letters above the columns indicate significant differences (*p* < .05).

Infrared drying is characterized by homogeneity of heating, high heat transfer rate, low heating time, low energy consumption, and improved food product quality (Aboud et al., 2019). Figure 4a,b demonstrate the influence of drying techniques on the total phenolic content and antioxidant capacity of fried falafels, respectively. The total phenolics contents of fried falafel samples prepared from hot‐air‐dried, infrared‐dried, and microwave‐dried ground sprouted chickpeas were 333.30, 428.27, and 198.87 μg GA/g dry, respectively. Infrared drying induced the release of covalently linked phenol compounds, resulting in higher antioxidants and phenolic compounds (Chatchavanthatri et al., 2021). Also, the antioxidant capacity of fried falafel samples prepared from hot‐air‐dried, infrared‐dried, and microwave‐dried ground sprouted chickpeas was 78.59%, 90.94%, and 42.24%, respectively. The total phenolics content and antioxidant capacity of fried falafels made from infrared‐dried ground sprouted chickpeas were significantly higher than those of the fried falafels prepared from hot‐air‐dried and microwave‐dried ground sprouted chickpeas (*p* < .05). Aboud et al. (2019) reported that the infrared drying achieved the highest antioxidant efficacy. Effects of parboiling and infrared drying on the quality of sprouted brown rice were studied by Chatchavanthatri et al. (2021). Their results showed that the infrared drying enhanced the bioactive compounds of non‐parboiled sprouted brown rice, including γ‐aminobutyric acid, α‐tocopherol, and total phenolic compounds, while γ‐oryzanol and antioxidant activity were comparable to brown rice.

### 3.4 Color of fried falafels

The frying process generates important changes at a superficial level in the materials (Coria‐Hernández et al., 2023). The effects of drying techniques of the ground sprouted chickpeas on the color parameters of fried falafel are reported in Table 3. The core color of the fried falafels was lighter and yellower than the surface, while the fried falafels’ surface color was redder than the core. The drying techniques did not have a significant impact on the surface and core lightness and yellowness indexes of the fried falafels (*p* > .05). The surface and core redness values of fried falafel made from hot‐air‐dried chickpea flour were significantly lower than those of the fried falafel made from infrared‐dried flour (*p* < .05). The mean values of lightness, redness, and yellowness of the fried falafels surface were between 43.11 and 47.48, 12.74 and 16.96, and 31.01 and 35.06, respectively. Furthermore, the mean values of lightness, redness, and yellowness of the fried falafels core ranged from 57.37 to 59.29, 3.66 to 6.88, and 40.50 to 43.31, respectively.

**TABLE 3:** Impact of drying techniques of the ground sprouted chickpeas on color parameters of fried falafel.

Drying method	Surface color indexes			Core color indexes		
	Lightness	Redness	Yellowness	Lightness	Redness	Yellowness
Hot‐air	44.76 ± 0.94^a^	12.74 ± 0.62^b^	31.01 ± 1.12^a^	58.15 ± 0.63^a^	3.66 ± 0.31^b^	40.50 ± 1.48^a^
Infrared	47.48 ± 3.59^a^	16.74 ± 0.51^a^	35.06 ± 3.94^a^	57.37 ± 0.94^a^	6.88 ± 0.26^a^	42.25 ± 1.91^a^
Microwave	43.11 ± 2.07^a^	16.96 ± 0.27^a^	33.27 ± 2.71^a^	59.29 ± 3.98^a^	4.14 ± 0.70^b^	43.31 ± 2.13^a^

*Note*: Data are shown as mean ± standard deviation (*N* = 3). According to the one‐way ANOVA and the Duncan post hoc test, different letters within columns indicate means that are significantly different at *p* < .05.

### 3.5 Volume and density of fried falafels

**FIGURE 5:**
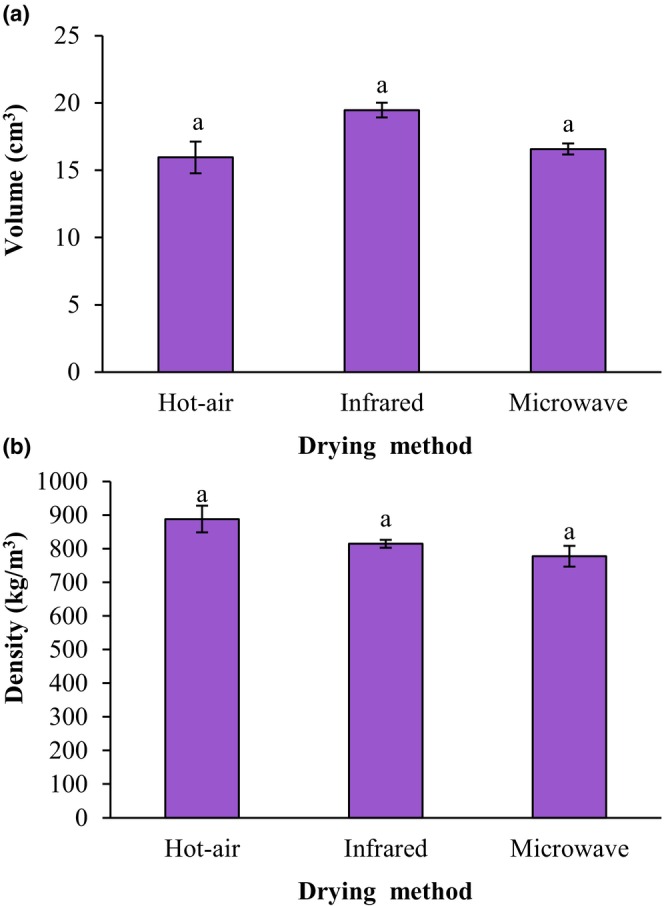
Impact of drying techniques of the ground sprouted chickpeas on volume (a) and density (b) of fried falafel. Data are shown as mean ± standard deviation (*N* = 3). According to the one‐way ANOVA and the Duncan post hoc test, different letters above the columns indicate significant differences (*p* < .05).

Figure 5 displays the effect of drying techniques of the ground sprouted chickpeas on the volume and density of fried falafels. In terms of volume, there was no statistically significant change between the fried falafel samples (*p* > .05). The highest volume was related to the samples prepared from the infrared‐dried sprouted chickpea. The volume of fried falafel samples prepared from hot‐air‐dried, infrared‐dried, and microwave‐dried ground sprouted chickpeas was 15.95 cm^3^, 19.47 cm^3^, and 15.67 cm^3^, respectively. The findings of this study confirmed that the drying techniques of the ground sprouted chickpeas did not have a significant influence on the density of the fried falafels (*p* > .05) and the mean values of fried falafels density were between 777.08 kg/m^3^ and 887.91 kg/m^3^.

### 3.6 Oil content of fried falafels

**FIGURE 6:**
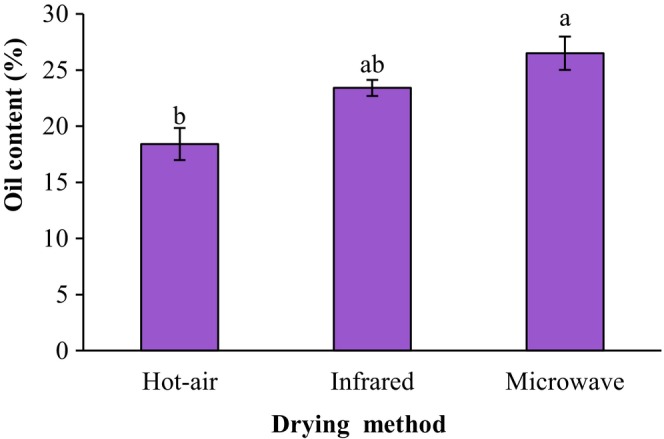
Impact of drying techniques of the ground sprouted chickpeas on oil content of fried falafel. Data are shown as mean ± standard deviation (*N* = 3). According to the one‐way ANOVA and the Duncan post hoc test, different letters above the columns indicate significant differences (*p* < .05).

During the frying process, the surface temperature of the raw material rises rapidly, causing water evaporation to form a dry layer on the surface of the food. Food products usually absorb a large amount of oil during frying (Chen et al., 2024). Figure 6 displays the effect of drying techniques of the ground sprouted chickpeas on the oil content of fried falafel. The lowest oil absorption was related to the sample prepared from the hot‐air‐dried sprouted chickpeas (*p* < .05). Oil absorption is closely related to the evaporation of water of samples, and the more water evaporation, the more oil absorption. During the microwave drying, water of ground sprouted chickpeas would evaporate from the interior of the material and generate a porous structure, which affected the oil uptake (Jia et al., 2018). The oil contents of fried falafel samples prepared from hot‐air‐dried, infrared‐dried, and microwave‐dried ground sprouted chickpeas were 18.42%, 23.42%, and 26.50%, respectively. The effect of pre‐drying, including hot‐air, vacuum microwave, and infrared, on the water and oil state breaking force of fried potato slices was investigated by Jia et al. (2018). Their finding showed that the drying method can effectively hinder the oil absorption of fried potato slices and have no negative effects on its breaking force.

### 3.7 Textural properties of fried falafels

**FIGURE 7:**
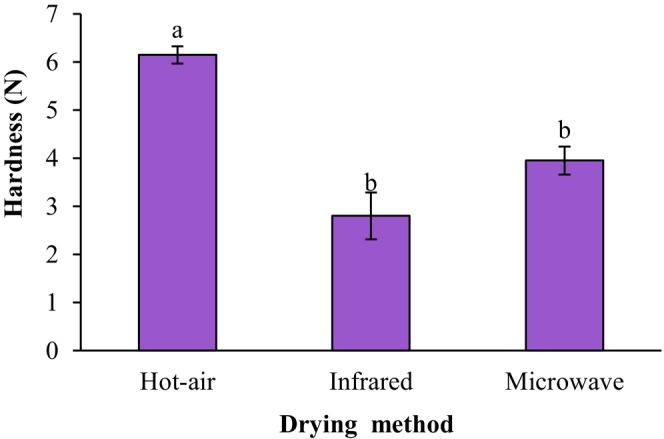
Impact of drying techniques of the ground sprouted chickpeas on texture hardness (puncture test) of fried falafel. Data are shown as mean ± standard deviation (*N* = 3). According to the one‐way ANOVA and the Duncan post hoc test, different letters above the columns indicate significant differences (*p* < .05).

Texture profile analysis directly influences consumer acceptability of the developed food products (Tukassar et al., 2023). The texture of the fried falafels was analyzed by means of the maximum force, the area of the curve, and the deformation time in the time–force curves measured by the puncture test corresponding to crust hardness (Li et al., 2022). Figure 7 shows the effect of drying techniques of the ground sprouted chickpeas on the texture hardness (puncture test) of fried falafel. Drying techniques of the ground sprouted chickpeas have a significant influence on the hardness of the fried falafels (*p* < .05). The lowest hardness values were related to the fried falafel prepared from the infrared‐dried sprouted chickpeas. The changes in crust hardness were related to water, oil content, and surface microstructure of fried samples (Li et al., 2022). The hardness of fried falafel samples prepared from hot‐air‐dried, infrared‐dried, and microwave‐dried ground sprouted chickpeas was 6.15 *N*, 2.80 *N*, and 3.95 *N*, respectively.

After performing the TPA test, the parameters of firmness, cohesiveness, springiness, and chewiness related to fried falafels prepared from sprouted chickpea flours were calculated and are reported in Table 4. In this study, the mean values of firmness, cohesiveness, springiness, and chewiness of the fried falafels ranged from 38.17 *N* to 91.56 *N*, 0.29 to 0.44, 0.48 to 0.58, and 5.38 *N* to 17.24 *N*, respectively.

**TABLE 4:** Impact of drying techniques of the ground sprouted chickpeas on textural properties (TPA test) of fried falafel.

Drying method	Firmness (*N*)	Cohesiveness	Springiness	Chewiness (*N*)
Hot‐air	56.73 ± 5.84^b^	0.44 ± 0.030^a^	0.58 ± 0.04^a^	14.50 ± 1.91^a^
Infrared	38.17 ± 7.67^c^	0.29 ± 0.026^b^	0.48 ± 0.02^b^	5.38 ± 1.29^b^
Microwave	91.56 ± 2.45^a^	0.39 ± 0.033^a^	0.48 ± 0.03^b^	17.24 ± 2.39^a^

*Note*: Data are shown as mean ± standard deviation (N = 3). According to the one‐way ANOVA and the Duncan post hoc test, different letters within columns indicate means that are significantly different at *p* < .05.

As can be seen from Table 4, there is a statistically significant change between fried falafel samples concerning firmness (*p* < .05). The lowest firmness was related to the fried falafel prepared from the infrared‐dried sprouted chickpeas.

The results of this research indicated that there was considerable difference among the fried falafel samples in terms of texture and cohesiveness (*p* < .05). The lowest cohesiveness was related to the fried falafel prepared from the infrared‐dried sprouted chickpeas.

The highest springiness values were related to the fried falafel prepared from the hot‐air‐dried sprouted chickpeas.

The findings of this research showed that the drying techniques of the ground sprouted chickpeas have a significant influence on the chewiness of the fried falafels (*p* < .05). The lowest chewiness value (5.38 *N*) was related to the fried falafel prepared from the infrared‐dried sprouted chickpeas.

### 3.8 Sensory evaluation of fried falafels

Deep‐frying process not only imparts a crispy texture, golden color, and unique fried flavor to food items but also triggers important changes, such as water vaporization, diffusion of oil, denaturation of proteins, and starch gelatinization, along with intricate interactions among various constituents (Li et al., 2024). Table 5 shows the effect of drying techniques of the ground sprouted chickpeas on the sensory attributes of fried falafel. The drying techniques of the ground sprouted chickpeas have a significant effect on the surface brightness, core brightness, and appearance acceptance of the fried falafels (*p* < .05). The highest surface brightness, core brightness, and appearance acceptance scores were related to the fried falafel prepared from the hot‐air‐dried sprouted chickpeas. The firmness and texture acceptance of fried falafels made from hot‐air‐dried sprouted chickpeas were significantly higher than those of fried falafels prepared from infrared‐ and microwave‐dried ground sprouted chickpeas (*p* < .05). The highest odor, flavor, and overall acceptance scores were related to fried falafel prepared from infrared‐dried sprouted chickpeas.

**TABLE 5:** Impact of drying techniques of the ground sprouted chickpeas on sensory attributes of fried falafel.

Drying method	Surface brightness	Core brightness	Appearance acceptance	Odor acceptance	Flavor acceptance	Firmness	Texture acceptance	Overall acceptance
Hot‐air	6.42 ± 0.75^a^	7.79 ± 0.61^a^	7.58 ± 1.04^a^	6.37 ± 1.09^b^	7.21 ± 0.61^a^	7.16 ± 0.74^a^	7.42 ± 1.09^a^	7.11 ± 0.64^a^
Infrared	5.37 ± 0.67^b^	6.53 ± 0.94^b^	7.21 ± 0.83^a^	7.53 ± 0.88^a^	7.58 ± 1.14^a^	6.26 ± 0.71^b^	6.47 ± 0.50^b^	7.26 ± 0.71^a^
Microwave	5.58 ± 0.94^b^	7.00 ± 1.21^b^	5.84 ± 0.81^b^	7.00 ± 0.73^a^	6.47 ± 0.99^b^	5.42 ± 0.94^c^	6.16 ± 0.87^b^	5.89 ± 0.72^b^

*Note*: Data are shown as mean ± standard deviation (*N* = 3). According to the one‐way ANOVA and the Duncan post hoc test, different letters within columns indicate means that are significantly different at *p* < .05.

## 4 CONCLUSION

In this work, the effect of different drying techniques, including hot‐air, infrared, and microwave, on the physicochemical characteristics of dried ground sprouted chickpeas was examined. The results of this work demonstrated that there was no statistically significant change in the ash content of dried ground sprouted chickpeas. Since the drying time of the ground sprouts in the infrared‐dryer was shorter than in the hot‐air and microwave dryers, more phenolic compounds remained in the prepared flour. The dried ground sprouts with infrared radiation had the lowest lightness, and the highest redness and yellowness indexes.

In addition, in this research, the effects of drying techniques of ground sprouted chickpeas on the physicochemical characteristics and sensory attributes of fried falafel were studied. The moisture content, total phenolics content, and antioxidant capacity of fried falafels made from infrared‐dried ground sprouted chickpeas were significantly higher than those of the fried falafels prepared from hot‐air‐ and microwave‐dried ground (*p* < .05). The lowest oil absorption was related to the sample prepared from the hot‐air‐dried sprouted chickpeas (*p* < .05). The lowest firmness was related to the fried falafel prepared from the infrared‐dried sprouted chickpeas. Due to the highest odor, flavor, and overall acceptance scores, high content of phenolic compounds, high antioxidant activity, high volume, low density, and low hardness, it is recommended to use the infrared‐dryer for drying ground sprouted chickpeas before producing fried falafel.

## AUTHOR CONTRIBUTIONS

**Kimia Goharpour:** Data curation (equal); formal analysis (equal); investigation (equal); software (equal). **Fakhreddin Salehi:** Conceptualization (equal); data curation (equal); formal analysis (equal); investigation (equal); methodology (equal); software (equal); supervision (equal); validation (equal); writing – original draft (equal); writing – review and editing (equal). **Amir Daraei Garmakhany:** Conceptualization (equal); data curation (equal); investigation (equal); methodology (equal); writing – original draft (equal).

## FUNDING INFORMATION

This work was supported by a grant from the Bu‐Ali Sina University, Hamedan, Iran (Grant No. 402174 to Fakhreddin Salehi).

## CONFLICT OF INTEREST STATEMENT

None.

## ETHICS STATEMENT

None.

## Data Availability

All data generated or analyzed during this research are included in this published manuscript.
